# Simulations of Attosecond Metallization in Quartz and Diamond Probed with Inner-Shell Transient Absorption Spectroscopy

**DOI:** 10.1021/acs.jpca.4c05137

**Published:** 2025-01-13

**Authors:** Lucas Kurkowski, Adonay Sissay, Mengqi Yang, Alexander Meyer, Kenneth Lopata

**Affiliations:** † Department of Chemistry, Louisiana State University, Baton Rouge, Louisiana 70803, United States; ‡ Center for Computation and Technology, Louisiana State University, Baton Rouge, Louisiana 70803, United States

## Abstract

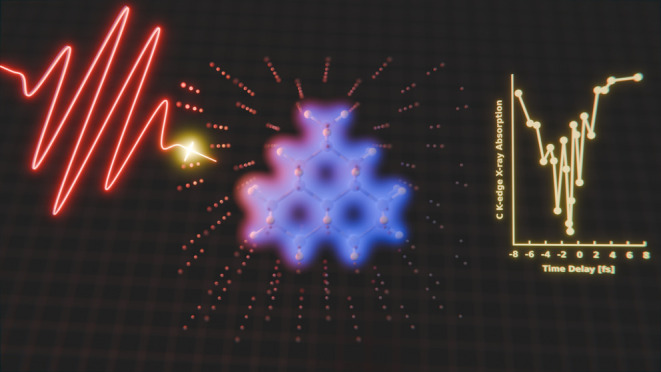

When dielectrics are hit with intense infrared (IR) laser pulses, transient metalization can occur. The initial attosecond dynamics behind this metallization are not entirely understood. Therefore, simulations are needed to understand this process and to help interpret experimental observations of it, such as with attosecond transient absorption (ATA). In this paper, we present first-principles simulations of ATA based on bulk-mimicking clusters and real-time time-dependent density functional theory (RT-TDDFT), with Koopmans-tuned range-separated hybrid functionals and Gaussian basis sets. Our method gives good agreement with the experiment for the breakdown threshold in silica and diamond. This breakdown voltage corresponds to a Keldysh parameter of approximately one and thus involves a transition to a regime where the dynamics are driven by tunneling. Pumping at an amplitude just below this value causes a mixture of multiphoton and tunneling excitations across the band gap to occur. The computed extreme ultraviolet and X-ray attosecond transient spectra also agree well with the experiment and show a decrease in optical density due to the transient population of the conduction band from the IR field. First-principles approaches such as this are valuable for interpreting the complicated modulations in a spectrum and for guiding future attosecond experiments on solids.

## 1 Introduction

Building on many of the same tools as gas-phase studies of atoms and molecules under strong fields, condensed matter systems have been the focus of much recent progress. These experiments and simulations have shed light on interesting processes such as band gap tunneling,^1−7^ carrier transport,^8,9^ dynamics at conical intersections,^10,11^ high harmonic generation,^12−15^ and strong field damage.^16−18^ With the advent of new ultrafast laser technology, it is now possible to probe dynamics on the subfemtosecond time scale. Over hundreds of attoseconds, the response is purely electronic, as the lattice motion and subsequent coupling to phonon modes occur much slower.

In the pursuit of more rapid optoelectronic switches, materials with wider band gaps, such as dielectrics, have been the focus of much study.^19−21^ Recent advancements in measuring some of these dynamics in dielectrics include energy transfer from ultrafast strong fields in silicon, diamond, and α-quartz;^22−24^ high harmonic generation of 200 attosecond pulses in α-quartz;^25^ electron scattering using attosecond streaking spectroscopy in dielectric nanoparticles and Si_3_N_4_.^26^ α-quartz, in particular, is one of the most well studied dielectric materials both experimentally and theoretically.^4,9,13,22,25,27−32^

In a seminal paper by Krausz and co-workers, α-quartz was shown to have a reversible transformation under strong fields. A single ultrashort pulse triggers an instantaneous polarization within the material, this response opens the door to attosecond control where optical signals and electric currents can be modulated.^28,30,33−36^ This property is foundational to applicability in the development of electronics and has been studied in several dielectrics.^5,24,30^ From a simulation standpoint, Stockman and Krausz used time-dependent Schrodinger equation (TDSE) with using fine-differences time-domain (FDTD) to reproduce a comparable result to experimental bulk measurements using a four band model.^28,30^ There they observed Wannier–Stark ladder formation that collapses the band gap, allowing for transfer from the valence band (VB) to the conduction band (CB). Similarly, time-dependent density functional theory (TDDFT)^37−42^ has also been used to capture α-quartz absorption.^43^ In addition, first-principles methods utilizing both RT-TDDFT and core-state-resolved Bloch equation models have been shown to accurately capture X-ray probed intra/interband dynamics at the CB on a number of materials.^44^

Simulations are critical to interpreting experiments but can be challenging in practice. Most such calculations rely on a computationally costly bulk structure and a grid- or plane-wave-based approach to solve the TDSE or TDDFT equations. Additionally, most well-traditional TDDFT exchange-correlation functionals are prone to self-interaction^45^ and charge delocalization error,^46^ especially in heavily driven systems. These errors are especially pathological in pure DFT functionals that essentially give incorrect description of the Coulomb potential and lead to electrons self-repelling themselves and thus an overdelocalization of charge density. For molecular studies, this has been shown to produce inaccurate calculations of charge transfer processes.^47−50^ For materials, computing optical gaps using DFT or TDDFT can be challenging, with global hybrid functionals such as B3LYP^51^ giving generally satisfactory gaps, but not as accurate as alternatives such as the meta-GGA SCAN^52,53^ and the range-separated HSE functional.^54^ Long-range corrected hybrid DFT functionals have been shown to have improved description of high energy Rydberg states.^49,50^ Furthermore, for X-ray absorption in solids^27^ and strong field ionization in molecules,^55,56^ we have shown that tuned range-separated hybrid (RSH) functionals with no experimental parametrization are superior to traditional functionals. For attosecond dynamics in molecules, global or range-separated hybrids have been shown to give improved dynamics over LDA or GGA functionals.^57,58^

Unfortunately, range-separated hybrid DFT calculations are generally unfeasible using plane wave or grid bases due to the difficulty evaluating the exact exchange operator. Moreover, these bases generally require pseudopotentials to describe the core electrons. Although core-hole pseudopotentials can be used for perturbative spectral calculations,^59^ they are unsuitable for explicit time-dependent simulations of core-level spectra, especially at the K-edge which requires an all-electron description. A remedy to both issues is to use atom-centered Gaussian basis sets since they allow for rapid computation of exact exchange and require no pseudopotentials. Simulating bulk systems with Gaussians can be done using bulk-mimicking clusters with chemical passivation and/or electrostatic embedding,^27,60−65^ but care must be taken to ensure cluster properties are converged with size. Moreover, these bases are incapable of describing high energy continuum states^66^ due to their poor support in the region of space far from the nuclei in the molecule/cluster. Moreover, steps must be taken to remove resulting nonphysical intruder peaks from inner-shell spectra.^27,67,68^ For strong-field ionization in molecules, this can be remedied to some degree by large diffuse basis sets.^55,69^ For such high energy and ionization processes, a more natural approach is to instead use TDDFT on a grid,^70,71^ plane waves with pseudopotentials,^72^ or a hybrid of localized and plane-wave bases.^73^

In this study, we utilize bulk-mimicking clusters and tuned RSHs to compute strong-field IR-induced transient metallization and the associated transient spectra in two solid-state systems. For these calculations, it is expected that Gaussian basis sets give a decent description of the dynamics and the spectra. The probe energies of interest are low in the X-ray near-edge (XANES) region, where the Gaussian basis set TDDFT is known to give good X-ray absorption spectra.^27,74,75^ Moreover, the strong-field interband processes in this system occur locally to the cluster and do not, for example, involve tunneling out of it.

First, we present results for SiO_2_, with the corresponding XUV attosecond transient spectra computed using real-time TDDFT. Spectra are validated against the experimental measurements.^33^ Second, we use proof-of-principle simulations to show that resonant K-edge attosecond transient absorption can measure an analogous process in diamond. Using attosecond X-ray pulses to probe transient metallization remains essentially unexplored and has numerous advantages over semicore probes, including inherent elemental specificity and localized nature of the excitations. This makes TR-XAS especially attractive for studying dynamics at and around dopants and defects.

## 2 Methods

### 2.1 Cluster Model and Electronic Structure

For this study, we used finite bulk-mimicking clusters with atom-centered basis sets and tuned range-separated hybrid DFT. All basis sets drawn from the Basis Set Exchange^76^ and all simulations were performed using the DFT and real-time time-dependent DFT (RT-TDDFT) module^77^ in NWChem,^78^ where time propagation is done in the canonical basis via the von Neumann equation:
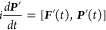
1where ***F***′ and ***P***′ denote the Fock and density matrices in the canonical basis. We use atomic units throughout unless otherwise noted.

Construction of the clusters involved truncating the periodic bulk structure and capping with hydrogens. To assess the convergence of the cluster, we used the optical gap (*E*_d_) of the structure computed via linear-response TDDFT. *E*_d_ is a simple indicator of the convergence of the optical properties without requiring calculating the full spectra or dynamics. Insensitivity of *E*_d_ to cluster size and convergence to the experimental value are indicators of a good bulk mimic. Convergence with respect to cluster size and basis set was assessed, as discussed in detail in Section 3.

From a DFT functional standpoint, tuned range-separated hybrid functionals^79^ are known to give improved strong-field dynamics in molecules^55,56^ and require no experimental parametrization. Range separation involves breaking up the exchange portion of the exchange-correlation potential into short- and long-range parts, with the short-range described using DFT exchange and long range described using HF exchange (see Supporting Information for details). We used a tuned version of the LC-PBE0 functional,^80^ where the attenuation parameter and global HF admixture parameter were tuned to satisfy Koopman’s theorem. This corresponds to minimizing the following target function
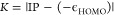
2where the IP is the first ionization potential computed at the DFT level via the energy difference of the cation and neutral system. Tuning the functional has the effect of reducing self-interaction error. This tuning was performed for the chosen cluster geometry and basis set.

### 2.2 Filtered Dipole Operator

Due to basis set limitations, nonphysical intruder peaks appear in real-time core-level spectra. These intruders, which involve transitions to poorly described virtual states,^68,81^ are especially problematic for systems with a high density of states. To remove them, when computing a spectrum, we use a filtered dipole operator that only includes transitions from particular core orbitals, e.g., Si 2p or C 1s. This is similar to windowing in linear response TDDFT, but instead, we filter the transition dipole matrix to exclude uninvolved molecular orbitals from the calculation of the spectrum, while leaving the interaction with the field unmodified. The details of this method are given in ref (68).

Briefly, before the time propagation begins the transition dipole matrix is converted to the basis of the ground state eigenvectors of the Fock matrix
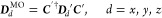
3where ***D***′ is the transition dipole matrix in the canonical basis and ***C***′ is the coefficient matrix in the canonical basis. Next, to remove undesired transitions from the spectrum, we zero the row and column of ***D***^MO^ containing any irrelevant orbitals. For instance, to obtain the transition dipole from only the *i*th orbital:
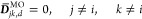
4This filtered***D*®**^MO^ is then used for calculating the time-dependent dipole moment **μ** during the propagation via
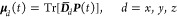
5where unprimed quantities denote the atomic orbital basis. For details regarding change of bases and validation for the case of SiO_2_, see ref (68). This technique is well-suited for deep inner-shell spectra, where the TDDFT character of the initial state of the transitions is essentially identical to the ground state molecular orbitals (e.g., 1s for K-edge).

### 2.3 Calculation of Attosecond Transient Spectra

To compute the transient spectra, the clusters are subjected to two time-delayed electric field pulses. The low frequency pump pulse transfers population from the VB to the CB, and the probe pulse causes core-level excitations (L/K-edge). Both fields have Hann envelopes defined by eq 6.
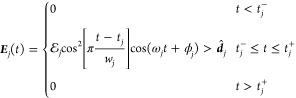
6where *j* = *P*, *p* for pump and probe, respectively.  denotes the amplitude of the pulse, *t*_*j*_ is the center of the pulse in time, *w*_*j*_ is the width in time, ω_*j*_ is the frequency, ϕ_*j*_ is the phase, and ***d̂***_*j*_ is the polarization. The start and end times for the pulses are given by  and , respectively. For all simulations, the pump was taken to be a few-cycle infrared pulse with ω_*P*_ = 0.057 au (800 nm), *w*_*P*_ = 550 au (13.3 fs), *t*_*P*_ = 350 au (8.5 fs), and . The amplitude, , varied by simulation. For the probe, the width was taken to be *w*_*p*_ = 5.0 au (0.12 fs), the amplitude was  au (0.0514 V/nm). The probe center time (*t*_*p*_) was varied, and thus the time delay between the pump and the probe. The probe center frequency (ω_*p*_) was chosen to be 4.1 au (11.1 nm, 111.6 eV, XUV) for the SiO_2_ study and 10.0 au (272.1 eV, 4.56 nm, X-ray) for diamond, such that the probe bandwidth covered the Si L-edge and C K-edge, respectively. The fields were coupled to the Fock matrix via the dipole approximation – ***D*** ·[***E***_*p*_(*t*) + ***E***_*P*_(*t*)].

The spectrum of the system can be computed from the Fourier transform of the dipole moment generated by subjecting the system to two pulses. The dipole strength function is given by
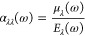
7
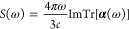
8where λ is the direction of the field (λ = *x*,*y*,*z*) and *c* is the speed of light in atomic units. The probe pulse is delayed in time relative to the center of the pump pulse in order to generate transient spectra. As our system does not incorporate any relaxation mechanisms, artificial dampening is applied to the dipole signal to broaden the spectral peaks. This was taken to be an exponential decay, starting from the beginning of the probe pulse
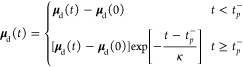
9where κ is the exponential decay time and **μ**_d_(0) is the dipole value at the beginning of the simulation.

To accelerate convergence (reduce simulation time), the dipole signal is transformed using Padé approximants.^67^ The use of Padé approximants on the electric field can be problematic due to the zero-values at the midpoint of the signal, which results in a singular inversion of the Toeplitz-symmetric matrix. To avoid this, the electric field signal is transformed with FFTs, and the resolution and frequency window of the dipole Padé transform are chosen to match the FFT over the spectral range of interest. Finally, the cluster is slightly asymmetric; thus, to generate a properly symmetrized (bulk-like) transient spectrum, the average of a spectrum from a positive and negatively polarized pump was taken. This is analogous to an orientational averaging for a molecular case. As an alternative, one could use a symmetrized cluster geometry. For a perfectly symmetric simulation (e.g., periodic boundary conditions), this would not be necessary.

### 2.4 Determination of Breakdown Voltage

When dielectrics are subjected to a field of sufficient intensity, the material is permanently altered, which results in a conduction band population that persists even after the field has passed. Determining this breakdown voltage (*E*_T_) directly from a simulation can be challenging, as subtle electronic structure errors can lead to large errors in the nonlinear response. As with molecular strong-field ionization, instead of directly using experimental pump intensities in a simulation, it is better to compute *E*_T_ self-consistently and pump with a laser intensity analogous to the experimental setup.^56^ In this work, we choose to pump in a self-consistently determined near-breakdown regime. Using a larger cluster and a functional that gives a more accurate TDDFT optical gap (if even tractable) would perhaps provide a somewhat different breakdown threshold. This is sidestepped, however, by pumping at an internally self-consistent Keldysh value, as computed from the optical gap of our cluster and the RSH DFT functional. In this way, both the experimental and simulated results should exhibit similar near-threshold dynamics. To compute *E*_T_, the CB population was summed over all virtual orbitals after the pump pulse was computed for a range of field amplitudes to find when the materials susceptibility to the electric field changes from one regime to another. This is related to the strong-field regime, as quantified by the Keldysh parameter, which for solid-state systems is calculated (in atomic units) via:
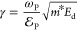
10where *E*_d_ is the optical gap. We opt to *E*_d_ instead of the (indirect) band gap *E*_g_, since the time scales involved are too fast for lattice coupling to occur. For both materials, we use an effective mass m* = 0.25*m*_e_, as was done in previous studies.^5,24^ Keldysh parameters of γ ≫ 1 correspond to the multiphoton excitations into the CB, whereas γ ≪ 1 corresponds to the tunneling regime. In the case of γ ≥ 1, both processes may occur to some extent.

## 3 Results and Discussion

### 3.1 Attosecond Band Dynamics in α-Quartz

First, we present simulations of intense infrared laser pulse-induced transient metallization of crystalline SiO_2_ (α-quartz). For this, we use a previously reported bulk-mimicking SiO_2_ cluster^27,68^ with the formula Si_5_O_16_H_12_ and *E*_d_ = 8.4 eV. This consists of a single central silicon atom connected to four bridging oxygens, which are in turn bonded to another shell of silicons bound to oxygens. The boundary oxygens are capped with hydrogens to chemically passivate the cluster. The basis set used for the central silicon atom was def2-TZVPPD, for the bridging oxygen def2-SVPD, and for the capping hydrogens, STO-3G was used. The Koopmans’ tuned range-separated hybrid functional LC-PBE0* is used, with the parameters α = 0.515, β = 0.485, and ζ = 0.101 au^–1^. For subsequent TDDFT simulations, we use a time step of Δ*t* = 0.2 au (0.005 fs) which was small enough to resolve core-level oscillations around 100 eV. All pump and probe pulses are linearly polarized and are applied in the *z*-axis, which due to symmetry was observed to give essentially the same unpumped spectrum as the average of the three polarizations. Simulations were run for a total of 1400 au (33.9 fs), followed by exponentially damping the time-dependent dipole moment μ(*t*) using a lifetime of κ = 75 au = 1.8 fs, which gives the peaks in the spectra a finite line width.

**Figure 1:**
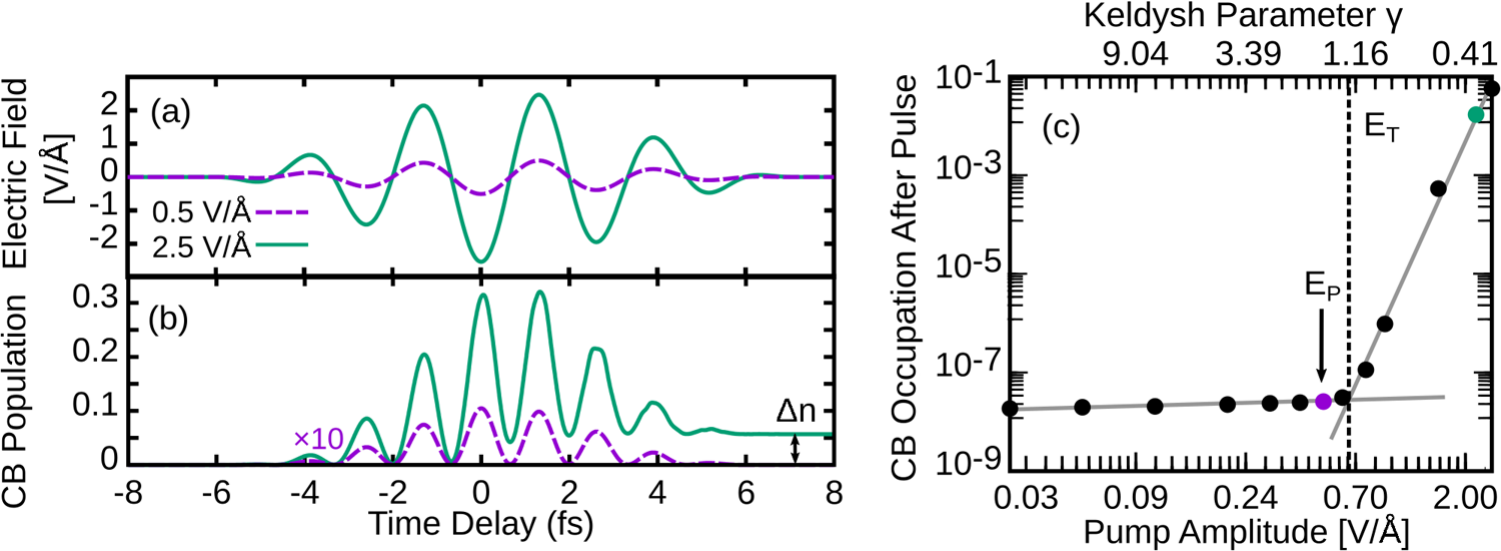
Strong-field infrared-induced transient conduction band population in SiO_2_ computed with LC-PBE0* and a bulk-mimicking cluster. (a) Applied pump electric fields with two different amplitudes. (b) The resulting conduction band occupations summed over all virtual orbitals for these two amplitudes, with Δn denoting the total occupation change after the pulse has passed. (c) The breakdown/threshold voltage (*E*_T_; vertical dashed line) is computed from the intersection of the linear fits, yielding a value of 0.62 V/Å. The corresponding Keldysh parameter shows that the *E*_T_ corresponds to a transition to a tunneling regime.

To compare to the experimental of transient metallization in this system,^19,33^ we use an infrared (NIR) pulse with a frequency of 1.55 eV and a pulse width of 13.3 fs. A sin^2^ envelope function is used to ensure zero-field amplitude at the beginning and end of the pulse. As outlined in the following section, the breakdown voltage for this system was then determined by computing the permanent CB population (Δ*n*) after the pulse as a function of pump amplitude. Figure 1a,b shows the results for the two representative intensities. The weaker field (purple), which has an amplitude of 0.5 V/Å (0.0097 au), corresponding to an intensity of 3.3 × 10^12^ W/cm^2^ results in a transient occupation during the pulse, primarily due to Stark shifting, where little CB population remains after the pulse. In the strong field case (2.5 V/Å, 8.3 × 10^13^ W/cm^2^, green curve), there is both a transient and permanent CB population. The permanent CB population is a measure of the irreversible change to the system due to field-induced transitions. Since these simulations do not include nuclear motion, the permanent population transferred to the CB cannot relax back down to the VB. At longer time scales, the electrons will couple to lattice degrees of freedom, leading to relaxation of the CB population over 60 or so femtoseconds,^5^ but this would require coupling to the lattice via Ehrenfest, surface hopping, or similar.

Figure 1c shows Δ*n* as a function of the pump amplitude. As shown via the Keldysh parameter, low pump amplitudes (γ ≫ 1) correspond to a regime where multiphoton excitations across the band gap barely populate the CB. As the amplitude increases and the Keldysh parameter becomes closer to 1, the amount of permanent CB population increases linearly in a logarithmic representation, until reaching a critical amplitude *E*_T_, after which the CB population dependence on field has a substantially larger exponential constant (log–linear slope). At this threshold voltage, the Keldysh parameter becomes ∼1.31. That is, the “knee” in Figure 1c corresponds to the field at which tunneling from VB to CB becomes increasingly dominant due to overlap between the bands and the dressing field. Loosely speaking, this transition to tunnel-dominated dynamics corresponds to the breakdown voltage that one measures experimentally. The intersection of a linear fit to these two regimes gives a breakdown voltage of 0.62 V/Å, as compared to the experimental breakdown voltage of *E*_T_ = 2.5 V/Å^33^ (breakdown intensity 8.3 × 10^13^ W/cm^2^) for a 1.58 eV (∼780 nm) pulse. Our underestimation of *E*_T_ is likely a consequence of the finite cluster model we are using.

Next, we present simulated transient Si L-edge XUV absorption for probing band dynamics in SiO_2_ resulting from IR pumping at just below the breakdown threshold, and validate our results against the experiment.^33^ We perform our dynamics using an electric field amplitude below our computed *E*_T_, such that our dynamics are consistent with the experimental, even if there is a difference in the value of *E*_T_. To this end, we use a pump amplitude of 0.5 V/Å (γ = 1.63), which is just below the computed *E*_T_ value of 0.62 V/Å, and loosely corresponds to the experimental pump amplitude of 2.1 V/Å (versus observed *E*_T_ = 2.5 V/Å). The Si L-edge transient spectra for this system for two specific time delays were previously reported, as a means of validating our method for removing intruder peaks from the spectrum.^68^ See Supporting Information for these spectra, which correspond to the field off, ramping up (τ = −3.5 fs), and at the maximum (τ = 0 fs). The transient SiO_2_ spectra were shifted by 1.3 eV to match the ground state XANES spectrum. The near-edge spectrum of silica has two dominant peaks at 106.7 and 109.0 eV, which correspond to the transitions from the core Si 2p to 3s* and 3p* states, respectively. As the pump is turned on we see a decrease in the OD at these two peaks, due to the corresponding CB states becoming occupied while the pump is on. The 2p → 3s peak also exhibits a blue shift with instantaneous field magnitude. As analyzed in detail for diamond (below), this results from both nonadiabatic population of the CB, as well as Stark shifting of the 3s band due to the strong field. The 2p → 3p peak shows a similar, but less pronounced, shift.

**Figure 2:**
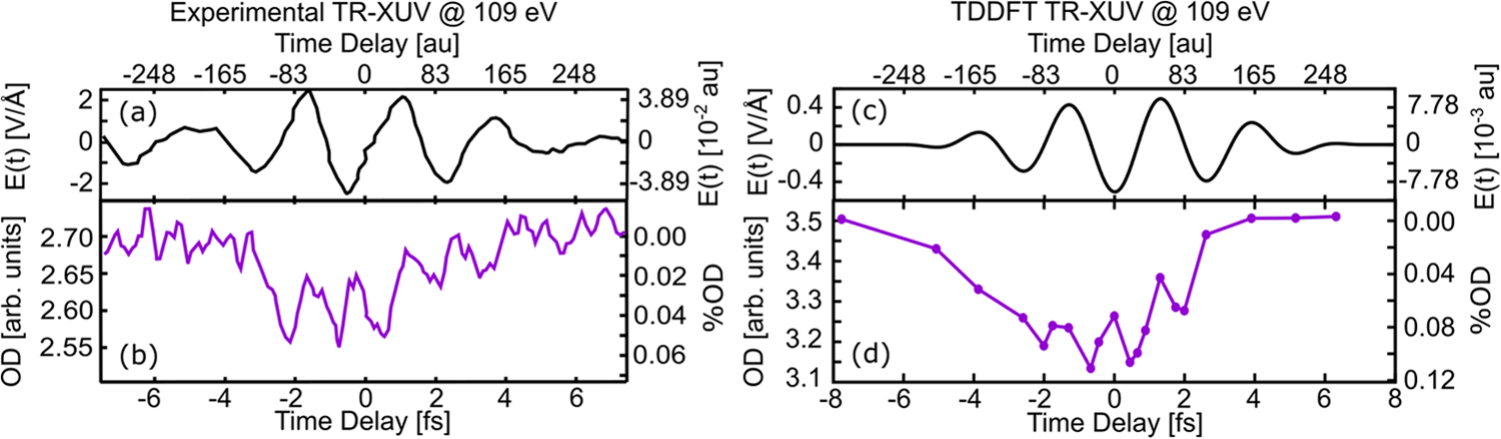
(a) Experimentally applied electric field and (b) experimentally measured transient XUV of SiO_2_ at the 109 eV Si L-edge peak for the case of a 2.5 V/Å infrared pump. (c, d) The corresponding TDDFT simulations for the case of 0.5 V/Å IR pump agree well with the experiments. The oscillatory reductions in the optical density (OD) are due to transient conduction band population caused by the IR pump, as well as instantaneous (Stark) effects. Experimental data were adapted from ref (33). Copyright 2013 Springer.

Looking at the modulations of the bright peak at 109 eV in particular, Figure 2 shows the optical densities as a function of the time delay for both the experiment and our TDDFT simulations. The TDDFT applied field (c) differs slightly in shape from the experimental one (a), which was reconstructed from an attosecond streaking measurement. Note that the TDDFT field amplitude is lower, such that both experiment and theory occur just below their respective breakdown thresholds, as discussed previously. The corresponding experiments and TDDFT OD modulations are shown in Figure 2c,d. As with the experiment, the computed OD at this peak exhibits an overall reversible decrease during the pulse. This is a consequence of increased occupation of the virtual states probed by this transition, with OD decreasing with increased occupation. This is qualitatively similar to the molecular case, where transient soft X-ray absorption decreases with increasing electron density around the absorbing atom.^82^ Additionally, there are oscillatory features during the pulse that occur at roughly double the frequency in both the experiment and TDDFT. The Keldysh parameter for this pump amplitude (γ = 1.63) indicates an intermediary regime where both tunneling and photon induced excitations can occur. The overall OD reduction where the electric field is zero is a result of transient CB population, since Stark effects do not contribute at these times. The asymmetry in the dynamics is a consequence of the carrier envelope phase of the pump. The oscillations in absorbance that occur align with the maxima of the field squared, with some amount of time-shift. The computed delay (∼500 as) of the response of the system, i.e., the field-squared maxima versus OD minima, is comparable but less than that of the experiment (∼850 as), likely due to finite size effect related to our cluster model and differences in pump intensity versus experiment. This can be understood as an underestimate of the time scale of electron localization due to the artificial confinement of the electrons within the finite cluster.

Overall, our computed TAS compares well with the experimental spectrum, albeit with an underestimated delay time and an overestimated OD modulation. The latter is likely due to the limited number of conduction band states as a result of the clusters and basis sets used to describe the system. These results show that TDDFT with optimally tuned range-separated functionals and finite clusters can capture attosecond metallization and the associated inner-shell transient XUV absorption spectra, without any experimental parametrization.

### 3.2 Attosecond Band Dynamics in Diamond

Next, we present results for attosecond dynamics in diamond, which is a prime candidate for attosecond transient absorption spectroscopy (ATAS) due to its wide band gap, technological utility, high optical transparency, and its large breakdown threshold. Previous attosecond strong field work has been done on diamond by Lucchini et al.^24^ where they utilized ATAS to observe the dynamical Franz-Keldysh effect in the conduction band using an XUV probe. To analyze their results, they simulated the transient absorption using grid-based TDDFT using a meta-GGA functional (PBE), and found the dominant role of intrasub-band transitions over interband transitions. In this section, with our bulk-mimicking cluster approach, we show that a similar effect can be probed using carbon K-edge attosecond transient absorption.

As with SiO_2_, to facilitate use of hybrid DFT functionals and to allow for inner-shell spectra, we use a bulk-mimicking cluster for diamond consisting of hydrogen passivated structures of carbon atoms. First we assessed convergence with cluster size. Clusters of nine different sizes were constructed: adamantane-like C_10_H_16_, diamantane-like C_14_H_20_, hexamantane-like C_30_H_36_ up to octamantane-like C_33_H_36_, and nonamantane-like C_34_H_36_, corresponding to increasing the system by one diamond unit cell while maintaining cluster compactness. Each of these structures was hydrogen capped and optimized using the def2-TZVP basis and B3LYP functional. The Supporting Information contains the *xyz* geometries for all of these clusters. The optical gap was then computed via linear response TDDFT (5 roots were calculated for each). Figure S2 shows the optical gap (*E*_d_) as a function of the cluster size. *E*_d_ is essentially converged at seven cells (heptamantane, C_30_H_34_), albeit it is underestimated versus experiment (7.1 eV^83^) by ∼1 eV due to B3LYP functional error. Instead of this large cluster, we instead chose tetramantane C_22_H_28_, around which *E*_d_ is insensitive to cluster size (triamantane C_18_H_24_, tetramantane, and pentamantane C_26_H_32_). In addition to speeding up the calculations, this cluster is more amenable to using tuned range-separated DFT functionals, which can be problematic for extended systems.

Building on this, the convergence of the tetramantane cluster with respect to the basis set was studied using the def2 family and the B3LYP functional. Due to a lack of size consistency with tuned LC functionals,^84,85^ we perform the cluster size convergence test using the B3LYP global hybrid. As shown in Table S1, *E*_d_ is essentially converged at the triple-ζ level, with removal of f-orbitals from carbon having no appreciable effect on the gap. This led to a cluster with the formula C_22_H_28_, the def2-TZVP (no f) for carbon atoms, and the def2-TZVP basis sets for hydrogens. This cluster and basis set were used for all subsequent diamond calculations. Finally, with this cluster and basis set, we then optimally tuned the LC-PBE0 functional via enforcement of Koopmans’ theorem for the first ionization energy. The resulting parameters were found to be α = 0.28, ζ = 0.15 au^–1^. This resulted in an optical gap of *E*_d_ = 7.49 eV, which agrees well with the experimental value of 7.1 eV.

**Figure 3:**
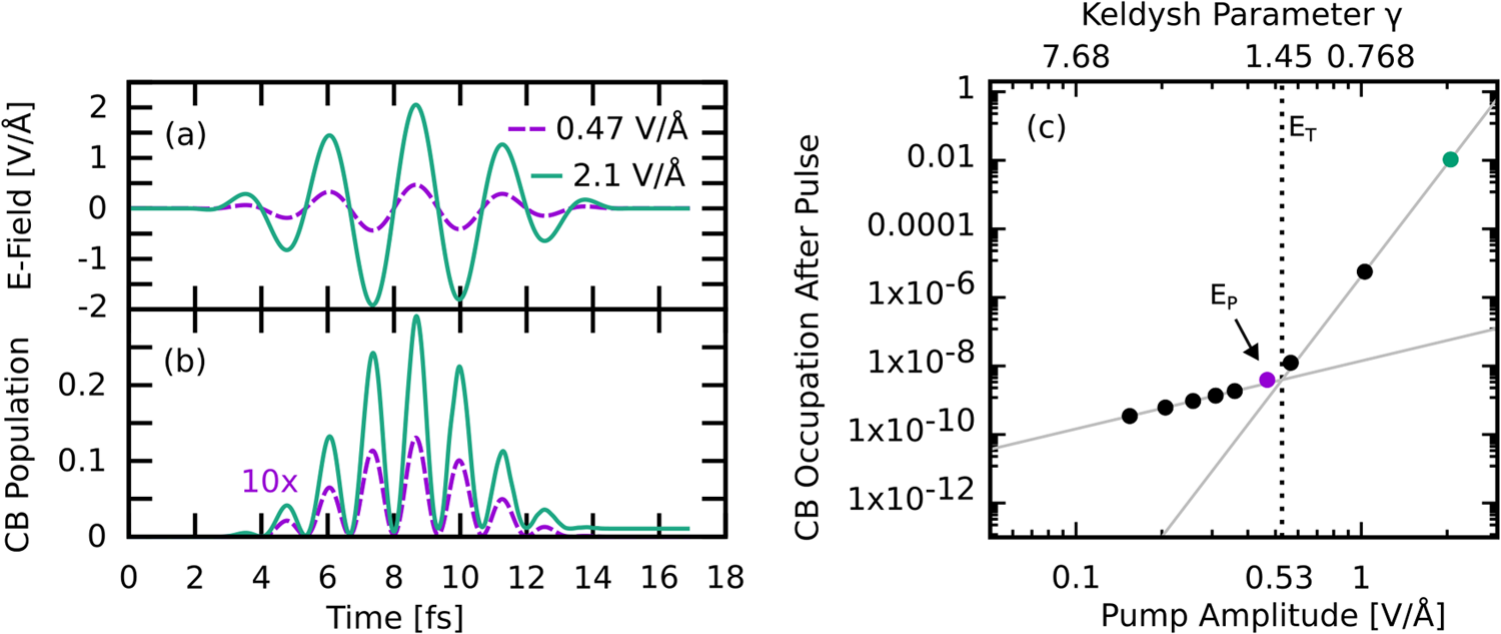
Simulated infrared-induced conduction band population in diamond. (a) Applied electric field for two amplitudes. (b) Computed conduction band populations for these amplitudes with the 0.47 V/Å population being multiplied, showing the nonlinearity of the response. (c) Permanent conduction band population in diamond as a function of pump amplitude. The dashed line at 0.53 V/Å denotes the breakdown voltage, where the Keldysh parameter switches from multiphoton to tunneling.

Before dynamics simulations, we first used RT-TDDFT to determine the breakdown voltage *E*_*T*_ for the diamond bulk mimic. As discussed previously, tuned range-separated hybrids are known to have improved behavior over LDA/GGA/global hybrids for strong-field processes.^41,86−88^ Charge density fitting with the def2-universal-JFIT auxiliary basis was used to accelerate the calculation. For this and all subsequent diamond simulations, the RT-TDDFT time step was taken to be Δ*t* = 0.1 au (0.0024 fs). As shown in Figure 3a,b, for low intensities (e.g., 0.47 V/Å (2.9 × 10^12^ W/cm^2^); purple curve), essentially all electrons go back to the VB after the pulse. For high intensities (e.g., 2.1 V/Å (5.8 × 10^13^ W/cm^2^); green curve), there is a permanent conduction band population, which corresponds to an irreversible electronic change in the material. Figure 3c shows a log/log plot of Δ*n* as a function of amplitude much like SiO_2_. In the case of diamond, the breakdown voltage was determined to be 0.53 V/Å (3.7 × 10^12^ W/cm^2^) which compares well with the experimental value of 0.73 V/Å (7.1 × 10^12^ W/cm^2^).^24^ This improved breakdown compared with the SiO_2_ case is likely due to the larger cluster size with a larger basis set, which provides a better description of the density of states. Physically, for amplitudes below *E*_T_ the IR pulse reversibly changes the band population via band tunneling and multiphoton excitations, whereas for amplitudes above *E*_T_, the strong field both reduces the band gap and creates a greater overlap between the VB and CB states, which results in drastic and permanent population transfer. As is the case with SiO_2_, there is no nuclear motion in these calculations, and thus the results are purely electronic and correspond to the attosecond-scale initial steps of dielectric breakdown.

**Figure 4:**
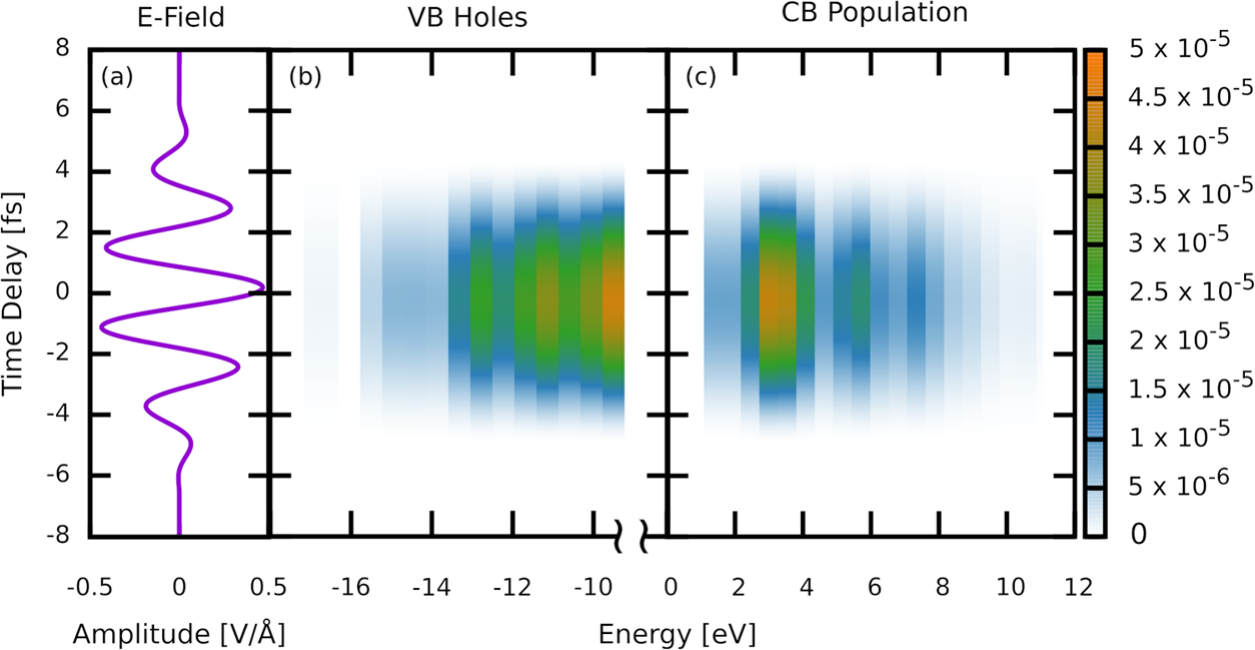
Time-dependent band populations in diamond subject to 0.47 V/Å infrared pulse. (a) Electric field applied to the system. (b) valence band hole density, and (c) conduction band population density, binned by state energy with a width of 0.02 au (0.54 eV) bins state energy. These populations were generated by fitting the occupations at the zero-field values to the pulse envelope (see text), and thus do not show Stark effects or oscillatory electron/hole dynamics that are occurring.

Next, we discuss the details of these dynamics for the case of a near-threshold pump, just below-breakdown (0.47 V/Å; *E*_p_, γ = 1.63). In the presence of a strong field, the energy levels are substantially Stark-shifted (see Supporting Information). Thus, when computing populations via projection onto a field-free Hamiltonian, there are apparent virtual populations, even when there is no excitation to the conduction band. To distinguish between Stark shifting and population transfer effects, for each orbital *k* we break the band/orbital occupation *N*_*k*_(*t*) into two components: a polarization part *p*_*k*_(*t*), which arises from shifting of the energy levels in the field, and a population part *n*_*k*_(*t*) due to transfer from VB → CB, with *N*_*k*_(*t*) = *p*_*k*_(*t*) + *n*_*k*_(*t*) Since there is no Stark effect when the field is zero, *n*_*k*_ = *N*_*k*_ at these times. To roughly quantify the populations during the pulse, we fit *n*_*k*_(*t*) to the same functional form as the envelope of the pulse. This assumes that the dynamics in the system roughly tracks the field envelope, which neglects any attosecond delay between the field and the electronic response. This analysis is done only for the purposes of quantifying the populations (Figure 4), and it has no impact on the transient absorption calculations. The polarization part, *p*_*k*_(*t*) is then the remainder of the occupation. See Figure S7 and associated text in the Supporting Information for details and plots. As our finite bulk-mimicking cluster does not truly have bands, we bin the populations by energy in the range of 0.02 au (0.544 eV). Note that the DFT virtual orbital energy levels are inaccurate, so the apparent band gap between the VB and CB is substantially greater than the TDDFT (optical) one.

Figure 4a shows the pump electric field, (b) the resulting energy-dependent VB hole population, and (c) the CB electron population. While the electric field is on, there is orbital localization that causes greater overlap between the VB and CB, and thus results in tunneling across the band gap. True Wannier–Stark localization cannot occur in our cluster due to boundary condition effects.^89^ The population reaches its maximum at the peak amplitude of the field and then returns back down to the VB once the field has passed. The remaining population after a pulse is too small to be visible with this scale. In this intermediate (Keldysh γ = 1.63) regime, the field induces a substantial transient and reversible population in the CB, and involves both multiphoton and tunneling processes. This change in population (∼10^–5^) is approximately 4 orders of magnitude greater than the permanent population induced by the field (∼10^–9^).

**Figure 5:**
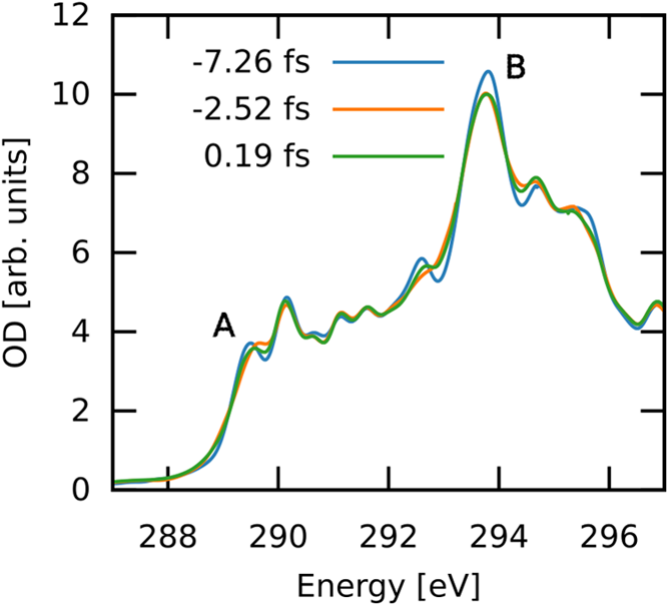
Carbon K-edge spectrum for diamond at three distinct time delays. The spectra were shifted +10.12 eV to match experimental energies. The three-time delays correspond to zero, rising, and maximum field amplitudes, respectively. The peaks A and B, both of which correspond to 1s → σ*, were chosen for detailed analysis of their OD modulations.

Finally, to determine how this transient metallization manifests in ATAS, we simulate the transient absorption at the diamond C K-edge. A pump with a range of positively and negatively time-delayed weak broadband probes with a center frequency of 10 au (272 eV), a sin^2^ envelope with a width of 5 au (121 as), and an amplitude 0.0001 au (0.0514 V/Å) was used. The simulations were run for a total of 750 au (15.7 fs) after the probe pulse, and the spectra were computed via Padé approximants. The dipole was exponentially damped with a time constant of κ = 110 au = 2.7 fs starting from the start time of the probe pulse *t*_*p*_^–^. See Section 2 for details. As shown in Figure S4, the *x*, *y*, and *z* polarized spectra were all similar, thus only *x*-polarized calculations were performed subsequently. Figure 5 shows the K-edge spectra at three particular time-delays, −7.26 fs when the pump field is not yet turned on, −2.52 fs when the field amplitude is a maximum during the ramp-up, and 0.19 fs, when the field is at the global maximum. Modulations are observed as a function of time delay, with both changes in the optical density, as well as subtle shifts in the peak positions. These responses encode information about the electron dynamics in the system. Two probe frequencies are labeled: Peak A (289.5 eV) corresponds to the rising edge, and Peak B (293.9 eV) is the brightest feature in this energy range of the near-edge spectrum. Both peaks have a predominantly 1s → σ* character.

**Figure 6:**
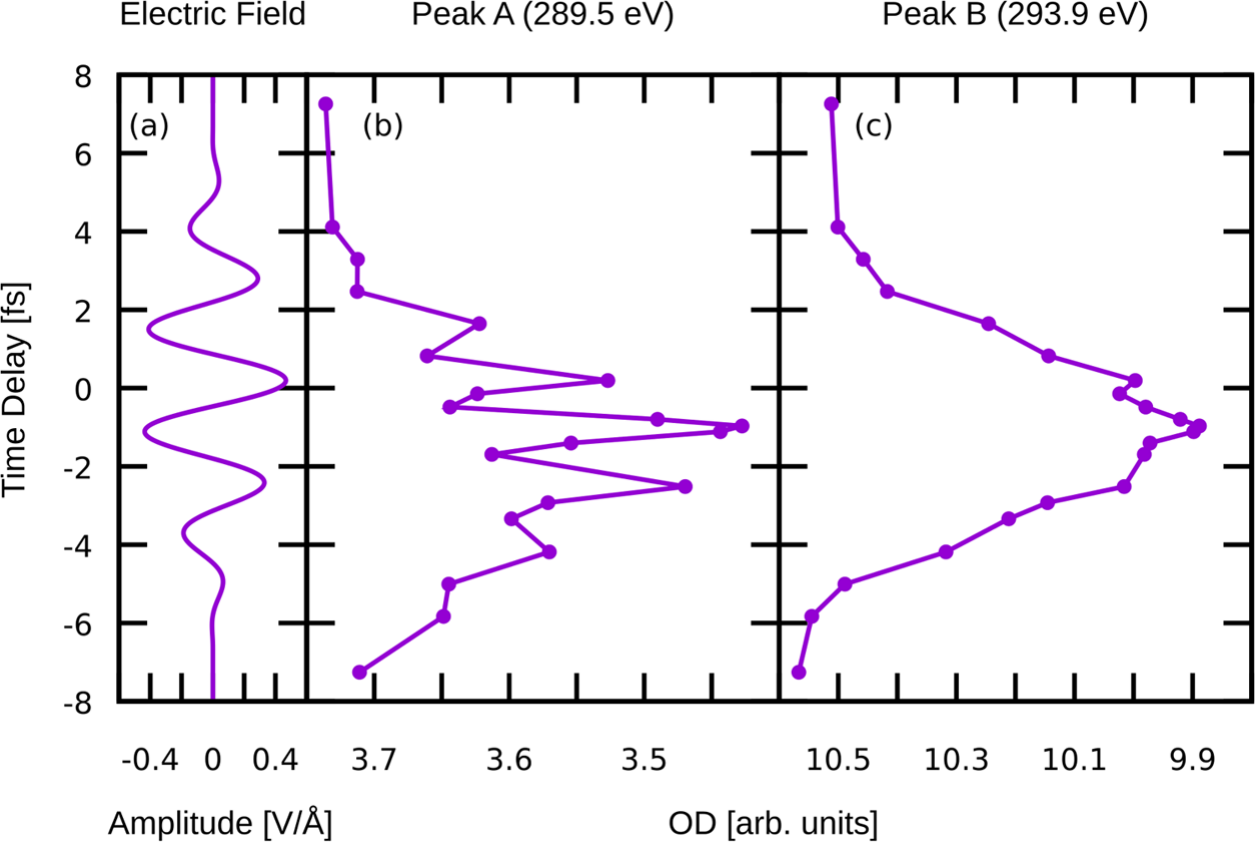
Transient optical densities evaluated at Peaks A and B. The broad overall OD reduction visible in both peaks corresponds to population transfer from the VB to CB, while the oscillations more visible in Peak A are due to Stark shifting.

Figure 6 shows the pump electric field, as well as the transient absorption at these two frequencies. The transient spectra exhibit two qualitatively different responses. There is a general decrease in the OD that tracks the field envelope; i.e., the conduction band population due to transient metallization (see Figure 4) reduces the absorption. This occurs via state blocking, where transitions to a state that is partially occupied are reduced. As with the SiO_2_ case, the asymmetry in the overall OD dip is due primarily to the asymmetry of the pump field (CEP) and the nonlinear nature of the process. The broad overall OD reduction at Peak B, which probes higher in the CB, is slightly narrower than that for Peak A. This is a consequence of higher field amplitudes being required to excite population this deep into the CB, i.e., more of the excitation is occurring near maximum values of the field.

In addition to this population effect, there is also band dressing that occurs which shifts the energy levels of the states in response to the applied oscillating field. This is shown clearly in Peak A in Figure 5, which shifts to higher frequency with larger instantaneous field magnitude, which reduces the observed OD at this specific energy. This field-induced OD reduction thus causes the transient spectra to oscillate at twice the frequency of the pump, consistent with the oscillations observed in the SiO_2_ and diamond experiments.^24,33^ For a detailed analysis of the Stark splitting of the states and the effect of electric field on the linear response C K-edge spectrum, see the Supporting Information. Our results show that there is appreciable Stark shifting at these field amplitudes, but the splitting and shifting of peaks does not completely describe the observed broadening and shifting. Thus, we conclude that both nonadiabatic (population) and Stark effects contribute meaningfully. This effect is more subtle in Peak B, where the population effect plays a much larger role in the OD reduction compared to the band dressing which can only be observed as slight oscillations visible near the OD minimum. This is potentially due to a reduced AC Stark polarizability for the states involved in Peak B, which are higher up in the CB.

Overall, these results demonstrate the viability and utility of using C K-edge ATXAS for these types of processes. In the case of Peak A (289.5 eV), there is an overall OD reduction of ∼7%, while in Peak B (293.9 eV) there is a reduction of ∼6%. This OD dip serves as a measure of the CB population in the states probed by the particular transition. This modulation is an order of magnitude greater than the population promoted to the CB (2.43 × 10^–4^ electrons, ∼0.015% change). That is, a small CB population change results in a significant OD modulation. TAS being sensitive to such small populations is beneficial, as lower pump amplitudes are still able to cause observable dynamics. Additionally, probing deeper into the CB (Peak B) exhibits less of an OD decrease as well as a slightly narrower overall dip and substantially less Stark shifts. Collectively, looking at different regions of the transient spectrum offers complementary information about attosecond dynamics. These differences between spectral features are also expected to be dependent on the pump intensity, giving a wealth of potential information in an attosecond experiment.

## 4 Conclusions

In summary, attosecond transient metallization of α-SiO_2_ and diamond via below-breakdown IR pump were simulated using RT-TDDFT with bulk-mimicking clusters, atom-centered Gaussian basis sets, and optimally tuned hybrid functionals. Unwanted transitions to nonphysical states were removed via a transition dipole filter. The corresponding XUV and X-ray transient absorption spectra were also computed, which yielded a relatively simple interpretation of the dynamics in terms of the instantaneous conduction band population. Using this method, the computed breakdown voltages show good agreement with experimental values and roughly coincide with the Keldysh parameter shifting from the multiphoton to the tunneling regime. The computed SiO_2_ transient spectra exhibits both the broad OD reduction as well as the oscillations at twice the pump frequency, as observed in experimental data from the literature.^33^

We expanded this approach to diamond, where C K-edge X-ray absorption was simulated during pumping just below the breakdown. The transient spectra show a response characteristically similar to that of silica involving a broad overall dip in the optical density as well as oscillations at twice the frequency of the field. The amplitude of the 2ω oscillations are related to the amount of Stark shifting occurring, while the broad overall decrease is a measure of the conduction band population. The results suggest that looking at different probe energies offers a more complete picture of the dynamics with different amounts of population and Stark modulations as you go higher in the spectrum. In particular, our diamond results suggest that in addition to XUV probes, using soft X-ray energies for measuring attosecond band dynamics in dielectrics is promising. Going forward, doped systems are especially interesting, and this type of simulation can play an important role since they require no inputs from the experiment. This makes them valuable for interpreting complex dynamics involved in transient spectra and for motivating future experiments.

## Supporting Information Available

Transient silica L-edge spectra; diamond cluster geometries and energies; basis set convergence data; range-separated hybrid functional discussion; effect of pump polarization on the carbon K-edge spectrum; and calculation of the Stark-shifted band-like states at the DFT and TDDFT levels (PDF)
