# Supplementary material for: Simulations of Attosecond Metallization in Quartz and Diamond Probed with Inner-Shell Transient Absorption Spectroscopy
Source: J Phys Chem A. 2025 Jan 13;129(3):650–60. doi: 10.1021/acs.jpca.4c05137 (PMC11770749; doi:10.1021/acs.jpca.4c05137)
Supplement: Supplementary file 1 — jp4c05137_si_001.pdf [file jp4c05137_si_001.pdf]

Supporting information for: Simulations of attosecond metallization in quartz and diamond probed with inner-shell transient absorption spectroscopy

Lucas Kurkowski,† Adonay Sissay,†,‡ Mengqi Yang,† Alexander Meyer,† and Kenneth Lopata*,†

†*Department of Chemistry, Louisiana State University*

‡*Present address: Northwestern State University of Louisiana, Natchitoches, LA*

¶*Center for Computation and Technology, Louisiana State University*

E-mail: klopata@lsu.edu

Contents

1 SiO_2 transient L-edge spectra **3**

2 Diamondoid size convergence **4**

3 Cluster geometries **5**

Adamantane ($C_{10}H_{16}$) 5

Diamantane ($C_{14}H_{20}$) 5

Triamantane ($C_{18}H_{24}$) 6

Tetramantane ($C_{22}H_{28}$) 7

Pentamantane ($C_{26}H_{32}$) 8

Hexamantane ($C_{30}H_{36}$) 10

Heptamantane ($C_{30}H_{34}$) 11

Octamantane ($C_{33}H_{36}$) 13

Nonamantane ($C_{34}H_{36}$) 15

4 Convergence with respect to basis sets **17**

5 Range-separated functional tuning **17**

6 Effect of pump polarization **18**

7 Stark-shifting of conduction band **19**

8 Fitting adiabatic population **21**

References **21**

1 SiO_2 transient L-edge spectra

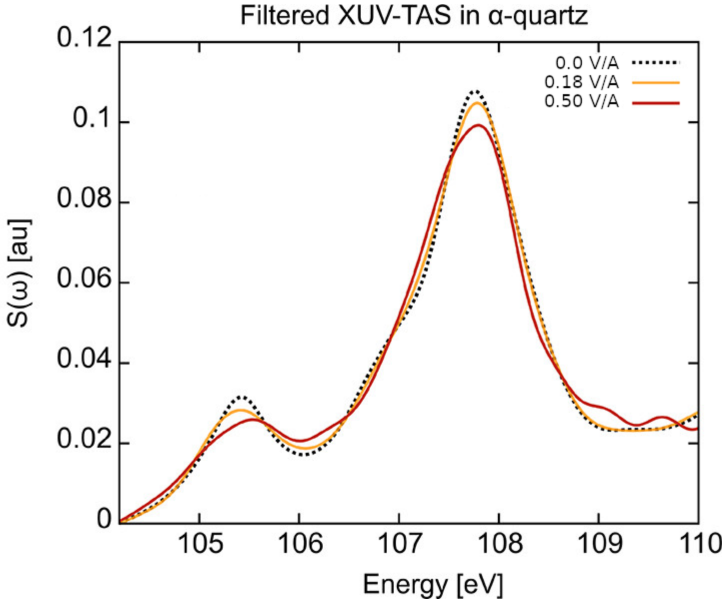

Figure S1: Three time delays for the L-edge spectrum of alpha-quartz under the effect of a strong field. Reproduced from Ref. 1. Copyright 2022 American Chemical Society.

Similar to Fig. 5 here are several time delays for the alpha-quartz cluster under the effect of the NIR/XUV pulse. The field amplitudes correspond to the field being off, ramping up, and its peak amplitude respectively. Note the spectra shown here are not shifted to match experiment, whereas the transient spectra in the main document are.

2 Diamondoid size convergence

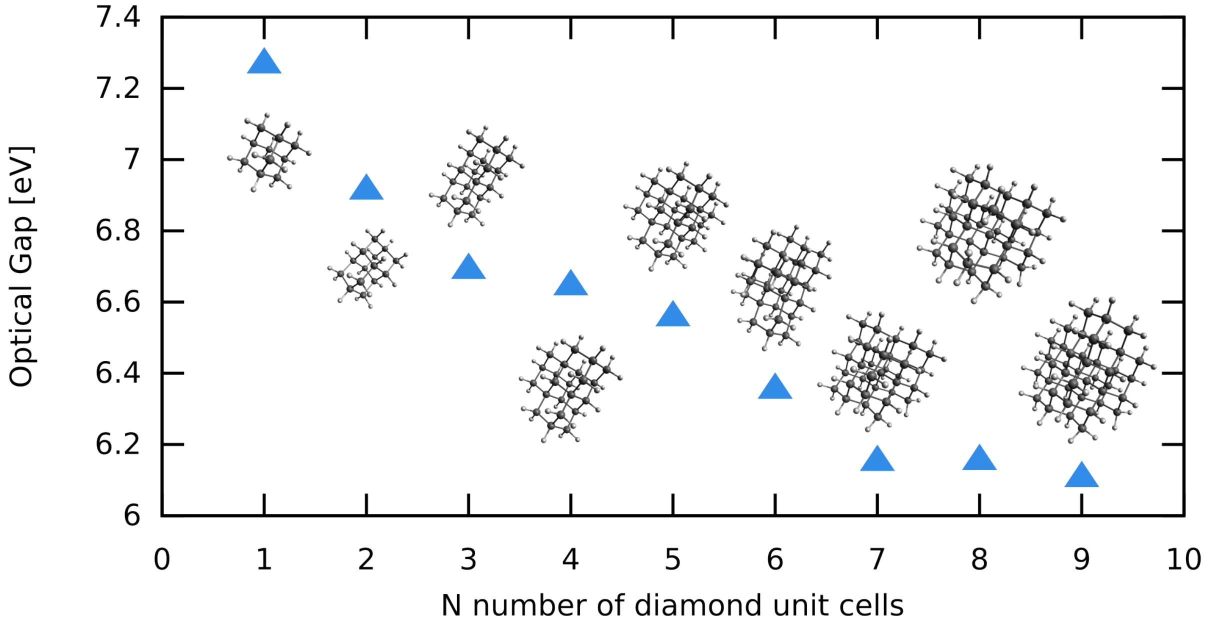

Figure S2: The linear response TDDFT optical gap for diamondoid-like structures as a function of size. All calculations used the B3LYP functional and the def2-TZVP basis. As the structure size increases the calculated optical gap value converges. Carbon atoms are represented in dark gray and the boundary passivating hydrogens in white.

To assess convergence with cluster size, we computed the linear response optical gap (E_d) for a range of geometries where we systematically added one unit cell in such a way to maintain cluster compactness. E_d for each cluster was computed using linear response TDDFT with the def2-TZVP basis set and the B3LYP functional. As shown in S2, there is a plateau between tri ($N = 3$) to pentamantane ($N = 5$), and after heptamantane ($N \geq 7$). Tetramantane ($N = 4$) was used for all subsequent calculations.

3 Cluster geometries

All of the following xyz geometries are in Angstroms.

Adamantane ($C_{10}H_{16}$)

H	-0.05224383	2.98882826	7.39038096
H	2.09087929	2.08243465	8.28993300
C	0.57946809	3.62054034	8.02209305
H	2.09088004	3.88837903	6.48398789
H	0.84730602	3.88838000	11.33944913
C	2.35871797	3.62054055	9.80134418
C	2.35871868	5.39979073	8.02209328
H	3.89682423	3.88837943	8.28993339
H	0.84730715	6.93789631	8.28993140
C	1.46909359	4.51016544	7.13621105
H	0.84730619	2.08243533	9.53350518
H	0.84730746	5.13195172	6.48398750
C	1.46909287	2.73465834	8.91171888
C	-0.30641419	4.51016588	8.91171776
H	2.09087944	6.93789652	9.53350435
H	2.09087878	5.13195245	11.33944918
H	-0.05224455	6.03150341	10.43305490
H	2.99042977	2.98882865	10.43305656
H	2.99043099	6.03150260	7.39038138
C	1.46909260	4.51016607	10.68722578
C	0.57946765	5.39979120	9.80134323
C	3.24460038	4.51016577	8.91171920
H	3.89682326	5.13195233	9.53350575
H	-0.95863790	3.88837977	9.53350392
H	-0.95863721	5.13195216	8.28993106
C	1.46909324	6.28567304	8.91171805

Diamantane ($C_{14}H_{20}$)

H	0.84825334	1.98921940	10.68739181
C	2.36688683	1.73829533	9.16856495
C	2.36699468	3.50946647	7.39719165
H	3.88849115	1.98788355	7.64711983
H	0.84815773	5.02823734	7.64826639
H	1.74334329	2.88952690	6.74456247
H	1.74305489	1.08570065	8.54882641
H	-0.05711963	2.89458782	8.55369985
C	1.46294070	4.41336704	11.84392548

C	2.34960359	5.29958239	9.18586999
C	2.34974796	3.52684326	10.95886492
C	1.46304839	6.18453818	10.07255205
C	-0.30838171	4.41339299	10.07259931
H	2.98653933	1.08547556	9.79223650
H	2.98658801	4.13317438	6.74437856
H	2.08659215	5.03330682	12.49655439
H	0.84339581	6.83735783	9.44888044
H	0.84334750	3.78965921	12.49673876
H	2.08688024	6.83713288	10.69229061
C	1.48033166	2.62325102	10.05524713
H	-0.05855590	5.93494984	11.59399718
C	0.57307249	5.30326806	10.96245516
C	0.57672761	3.52683002	9.18591339
C	3.25686280	2.61956535	8.27866187
C	3.25320768	4.39600356	10.05520375
H	4.79109109	2.88974837	9.79224184
H	4.79111210	4.13317836	8.54895977
H	2.98177767	2.89459620	11.59285058
H	2.98168203	5.93361396	8.55372531
H	3.88705476	5.02824583	10.68741734
C	4.13831697	3.50944058	9.16851778
H	-0.96117690	3.78965525	10.69215724
H	-0.96115565	5.03308533	9.44887523
C	1.48018735	4.39599028	8.28225208

Triamantane ($C_{18}H_{24}$)

H	-0.10116568	2.93991675	9.73541485
C	1.43480982	2.67278673	8.23730969
C	1.41764999	4.45873197	6.45286958
H	2.93650314	2.93987945	6.69931682
H	1.69184543	1.13708053	9.74634565
H	-0.10120342	5.97758437	6.69931572
H	0.79593814	3.83701963	5.79979403
H	1.68974378	2.94325167	11.54868680
H	0.80029358	2.03899949	7.60707264
H	-1.00208236	3.84137462	7.60707186
C	0.52117633	5.35520401	10.89463754
C	3.20502982	2.67135254	10.01676084
C	1.40048903	6.24467715	8.23730937
C	1.41764881	4.45873204	10.02845511
H	4.74317965	2.93788686	8.50629599
H	3.83493737	2.04144528	10.65196692

C	0.51751348	7.12959740	9.12687314
C	-1.25321654	5.35886646	9.12687279
C	2.31778524	1.78786687	9.12687399
H	2.03936260	5.08044462	5.79979503
H	2.93849744	1.13320342	8.50629495
H	1.14555376	5.97421303	11.54868594
H	-0.10319823	7.78426103	8.50629384
H	-0.09783260	4.73082639	11.54868580
H	1.14345332	7.78038340	9.74634514
C	0.53388645	3.57496885	9.10440255
H	-0.99963955	6.87601937	10.65196522
C	-0.36973177	6.24611197	10.01675954
C	-0.36829578	4.47589141	8.23730898
C	2.30451106	3.57187089	7.33313115
C	2.30141163	5.34249507	9.10440295
H	2.93312975	4.18663872	11.54868620
C	4.08851497	3.55859816	9.12687460
H	3.83738109	5.07608957	7.60707312
C	2.31412116	3.56226052	10.89463819
H	2.03500587	6.87846395	7.60707250
H	4.73929987	4.18453891	9.74634678
H	2.93646329	5.97754719	9.73541567
C	3.20359444	4.44157260	8.23731002
H	-1.90400230	4.73292623	9.74634457
H	-1.90788029	5.97957794	8.50629340
C	0.53078819	5.34559294	7.33313052

Tetramantane ($C_{22}H_{28}$)

H	-1.00192174	2.03877797	5.80323808
H	1.14394975	1.12822380	6.71146696
C	-0.37167959	2.67083675	6.43874467
H	1.13541187	2.93578333	4.89008715
H	-0.09649120	2.94550018	9.74556748
C	1.42278328	2.65654798	8.22705403
C	1.40461550	4.44501695	6.43914905
H	2.94202329	2.93684366	6.70725090
H	1.69332176	1.13178115	9.74329003
H	-0.10529308	5.98315331	6.70672746
C	0.51974852	3.56176127	5.54675813
H	1.69416177	2.94832389	11.56120413
H	-0.09365059	1.13215793	7.95611745

H	-0.10576245	4.17618303	4.88919177
C	0.52369650	1.79183930	7.33030310
C	-1.25015838	3.56492338	7.33027695
C	0.52310875	5.35946517	10.89884776
C	3.21064259	2.67045565	10.02096507
C	1.40486806	6.24977411	8.24449757
C	1.42391716	4.46420722	10.03413431
H	4.75942887	2.93589790	8.51397962
H	3.84630440	2.03877258	10.65124310
C	0.52135718	7.14282410	9.12808364
C	-1.25005485	5.35910183	9.12546089
C	2.31875572	1.79163621	9.12584393
H	2.04361785	5.08610797	5.81211759
H	2.93813631	1.12808529	8.50584961
H	1.14316763	5.97769719	11.56370416
H	-0.10600368	7.79900916	8.51357316
H	-0.09289934	4.73373805	11.55974497
H	1.13553224	7.79947767	9.75492518
C	0.53827693	3.58053364	9.11016570
H	-1.00223730	6.88524654	10.65102811
C	-0.37014719	6.25108280	10.01881296
C	-0.38503586	4.46508263	8.22514738
C	2.31245277	3.58215188	7.33654525
C	2.31413948	5.35376272	9.10795974
H	2.93771694	4.18411596	11.56415932
C	4.10218960	3.56122223	9.12971242
H	3.83702269	5.08533757	7.60469664
C	2.31868412	3.56453590	10.89953496
H	2.04512273	6.87646100	7.60431456
H	4.75990980	4.17691628	9.75407141
H	2.94303044	5.98237802	9.75393990
C	3.21060792	4.44464194	8.24468571
H	-1.91211536	2.94860604	7.95473491
H	-1.91543457	4.18460537	6.71215347
H	-1.91138685	4.73383508	9.74152818
H	-1.91527252	5.97814502	8.50659015
C	0.54110214	5.35440543	7.33477135

Pentamantane ($C_{26}H_{32}$)

C	2.92657996	6.12095560	6.31275501
C	2.00568855	5.19819350	5.50389966
C	3.86981354	5.26958968	7.17271372

C	2.07431182	7.01453770	7.22351341
C	-1.35528848	5.24239779	9.25686468
C	0.32833298	5.20687218	7.38275562
C	3.06862029	4.38733991	8.13986175
C	2.32761100	3.55441418	10.87373092
C	3.99674409	3.52755466	9.00935118
C	2.18641268	5.28049728	9.04736945
C	2.11801599	3.46815722	7.33295185
C	-0.52533518	4.33564411	8.33769156
C	-0.43999085	6.16819608	10.06952087
C	0.45215893	3.49119795	9.19136474
C	2.25704007	1.73443489	9.15480121
C	0.38309955	7.03848715	9.11061028
C	1.36970465	4.41135508	10.03486848
C	1.24305741	6.16073549	8.19061352
C	3.17808781	2.65356829	9.96822570
C	0.50989348	5.31962236	10.92513404
C	-1.42537179	3.40205275	7.51576941
C	0.36950149	1.65951621	7.46470565
C	0.24474755	3.38087382	5.65088340
C	1.17328081	4.31283030	6.44186077
C	1.29948448	2.56271111	8.28700150
C	-0.57850055	2.50461485	6.60406568
H	-0.35019805	5.81443694	6.76783711
H	1.75365016	2.94543695	11.58080557
H	2.97461580	4.20652457	11.47118427
H	4.62333492	2.89979628	8.36591468
H	4.67521278	4.17653825	9.57421950
H	2.84456923	5.94121725	9.62865962
H	2.72637752	2.82539257	6.68144771
H	-0.13653274	2.86553327	9.87670534
H	1.67953906	1.08879557	9.82579566
H	2.85246820	1.07316948	8.51539134
H	1.02888908	7.71864044	9.67704005
H	-0.28065128	7.66500801	8.50440891
H	3.85367813	2.04885325	10.58074789
H	1.16040407	5.96753718	11.52329617
H	-0.06136871	4.70726248	11.63154739
H	-2.12413683	3.99777134	6.91802757
H	-2.03159905	2.78894670	8.19204091
H	-0.20572022	1.01498372	8.13874725
H	0.96823992	0.99740733	6.82938194
H	0.83995642	2.75189741	4.97954619
H	-0.42122643	3.97724490	5.01721726
H	-1.23409336	1.84649752	6.02578923

H	3.51220687	6.74414142	5.63026194
H	4.54880758	5.91550357	7.74042123
H	4.49602446	4.63781859	6.53281041
H	2.59726875	4.56618810	4.83214210
H	1.33643293	5.79161630	4.87083886
H	1.40683369	7.64050607	6.62077719
H	2.71653223	7.69531049	7.79333375
H	-2.05400690	5.83459075	8.65565286
H	-1.96155640	4.62582242	9.92991086
H	-1.04588541	6.80789857	10.71825428

Hexamantane ($C_{30}H_{36}$)

C	5.22999071	7.63551954	8.50248097
C	5.92988773	6.79220693	7.42477512
C	4.44616354	6.72009980	9.45397952
C	4.25493282	8.60583679	7.82812950
C	4.54530973	4.38615529	4.69452634
C	3.75723672	3.43721154	5.61287577
C	3.57717272	5.37972845	4.04219013
C	5.59118063	5.16002447	5.50758050
C	1.08775185	2.56757826	6.93769076
C	4.90978562	5.95706599	6.63004831
C	3.21420550	7.79662596	7.05197088
C	3.88919741	6.93737040	5.97534585
C	2.85134697	6.16259850	5.14342252
C	1.37695136	6.08280063	7.19858107
C	4.11720687	4.99641182	7.58546854
C	3.51828798	3.89348302	10.30005055
C	5.02243446	3.95502334	8.29829020
C	3.41324231	5.87064866	8.68180204
C	3.03156021	4.21712763	6.74304615
C	0.56734561	5.17761432	8.13792341
C	1.54409658	4.19216634	8.79214015
C	3.13406642	2.29308784	8.41788446
C	2.61658668	4.92807763	9.61515220
C	2.43809731	6.85364775	7.98251208
C	4.20276671	3.04157883	9.22491494
C	-0.52659909	4.42738295	7.37598332
C	0.90871549	4.49633910	5.33372545
C	2.03985805	5.23649174	6.08051401
C	2.20809415	3.29105392	7.70653212
C	0.12938148	3.57606598	6.28432253

H	3.91388670	7.32641867	10.19590679
H	5.14121887	6.09609477	10.01531145
H	5.97868094	8.19576062	9.07105844
H	6.68704564	6.14530282	7.87578236
H	6.46443239	7.45069354	6.73114285
H	4.79122547	9.27891186	7.15103800
H	3.76326194	9.23288446	8.57910441
H	2.50502535	8.47892431	6.56855032
H	0.11138546	5.79705169	8.91929512
H	1.93917488	7.44737835	8.75990950
H	4.43512129	2.69172591	6.02612437
H	3.04031409	2.87752218	5.01236016
H	5.04724598	3.79724046	3.92034527
H	4.12694297	6.07668713	3.40128642
H	2.86944930	4.85714631	3.39488322
H	6.13140062	5.85283971	4.85292088
H	6.34114275	4.47893026	5.91839463
H	2.16104528	6.88185392	4.68510092
H	2.11676814	5.53794772	10.37811259
H	0.53314322	1.93171465	7.63668458
H	1.51032506	1.89669866	6.18496558
H	-0.63879298	3.03761166	5.72080374
H	4.45010021	7.59552038	5.29929281
H	-1.23250308	5.13656749	6.93147448
H	-1.09866939	3.79488323	8.06284757
H	5.79298170	4.45502321	8.88488625
H	4.26177510	4.37592011	10.93839766
H	3.59957923	1.61645803	7.69642144
H	4.87506896	2.31927745	9.69864025
H	5.55909636	3.34383109	7.57412365
H	0.23067501	5.24143948	4.90189221
H	2.91249462	3.25881923	10.95498546
H	0.69889790	6.80313219	6.72202396
H	2.53666536	1.66422009	9.08726361
H	0.98202903	3.53515033	9.46832887
H	1.29281598	3.91484356	4.49594146

Heptamantane ($C_{30}H_{34}$)

C	2.60953555	4.62655006	2.65738109
C	1.81926253	5.40333095	7.29402700
C	4.43365166	2.69000803	6.27374870

C	2.61862059	6.31492527	4.55493589
C	2.66879595	4.49667505	6.37996769
C	4.39286756	6.35124608	2.74320845
C	1.77435831	7.18419309	5.52292929
C	2.76545515	6.24534654	8.16720993
C	2.71772377	8.05791397	6.38018236
C	0.91675486	6.31765523	6.45679180
C	1.74223147	5.49170438	3.58629390
C	3.49165228	3.68709936	3.49542463
C	3.52211381	5.37249802	5.41490605
C	4.44648088	8.08038512	4.58247190
C	4.50733174	4.44822168	8.07660103
C	5.27916769	3.56834934	5.34021701
C	3.60547507	7.13409308	7.23562895
C	5.27960428	5.42780359	3.59871366
C	3.59224013	3.57444022	7.20143415
C	5.34921443	7.14346966	5.40190043
C	3.59222158	8.95383171	5.50212063
C	5.37512505	5.30822970	7.14344200
C	3.54331043	7.21934659	3.68925486
C	7.10426656	5.33036576	5.31969372
C	6.22525342	4.43872067	6.19747380
C	4.47453323	6.25030287	6.30801343
C	6.21388518	6.25686657	4.49046764
C	3.67468502	5.33953938	9.00247364
C	4.39020031	4.48717153	4.46273837
C	3.50117542	5.53468006	1.80819762
H	3.79013803	2.01506255	5.70522669
H	5.09535628	2.05296995	6.87012984
H	2.86070914	2.97787809	4.03143843
H	4.12962866	3.08994506	2.83401233
H	1.98839506	3.86636821	5.80339603
H	1.13383763	6.18039208	2.98909604
H	1.04049709	4.85565779	4.12612334
H	5.04016574	7.00873678	2.15093367
H	4.11403660	4.93776221	1.12473196
H	2.88937972	6.20039723	1.19043328
H	1.96033140	4.02985987	2.00912249
H	3.07292160	4.72222835	9.67815253
H	4.33400068	5.94693002	9.63182422
H	1.19977036	4.77090090	7.94065650
H	2.17140451	6.88317592	8.83187621
H	2.97348212	2.94105940	7.84766247
H	5.15781439	3.80137514	8.67654239
H	0.19350465	5.72502932	5.89171321

H	0.33170501	6.96614569	7.11797326
H	4.27637230	7.75382979	7.84533350
H	6.04656341	5.92767085	7.75284646
H	1.12319614	7.83803647	4.92989446
H	2.10899979	8.68056049	7.04590603
H	5.88040106	2.92960923	4.68151085
H	6.85987744	3.77732060	6.79852489
H	2.96713725	9.62719151	4.90589167
H	4.23093157	9.58546071	6.12866352
H	7.78654345	5.91680232	5.94427050
H	7.72661184	4.71725471	4.65906166
H	6.00549518	7.74713623	6.04249440
H	2.90524599	7.87998861	3.08807403
H	5.07306279	8.71646354	3.94635041
H	5.88753790	4.80196731	2.93251675
H	6.83724769	6.89637555	3.85456438

Octamantane ($C_{33}H_{36}$)

C	3.52815286	5.47036204	1.82461712
C	4.39132133	4.51140070	4.46505511
C	3.64924152	5.30300406	8.97495274
C	6.21574044	6.26720622	4.49021711
C	4.46277342	6.25563025	6.30495340
C	6.23646089	4.46468803	6.19945618
C	7.11495106	5.35640579	5.32370928
C	3.56856969	7.24475558	3.67709898
C	5.37009292	5.31975342	7.13845466
C	3.60995787	8.96374005	5.50671620
C	5.34914335	7.14842909	5.40444768
C	3.60997819	3.56853265	7.16100962
C	5.28236457	5.43472493	3.59569028
C	3.59348335	7.12739771	7.23505370
C	5.30412817	3.60454434	5.33062580
C	4.50814762	4.43778999	8.04876193
C	4.46734782	8.09486919	4.58233743
C	3.51078469	5.38581406	5.39845537
C	3.42012590	3.67695226	3.59579262
C	1.73981481	5.62325602	3.50135203
C	0.91714763	6.33557373	6.42974961
C	2.72479289	8.07075536	6.38091489

C	2.74511850	6.21423565	8.14007390
C	1.78150841	7.21468882	5.51195548
C	4.40135441	6.34510303	2.73367825
C	2.64189662	4.46035490	6.33095429
C	2.62110068	6.35644417	4.53435701
C	4.44285904	2.68478605	6.20271715
C	1.80125712	5.38158289	7.24893355
C	2.58499576	4.62414070	2.69507295
C	3.57091378	1.81384578	5.28871412
C	2.60667583	2.71651794	4.50220931
C	1.76421360	3.45603390	5.55393040
H	6.83135709	6.91278427	3.85284325
H	5.89186994	4.80109137	2.93778222
H	5.10559454	8.73491421	3.96185876
H	2.94404616	7.91464215	3.07340318
H	6.00496699	7.74181956	6.05463598
H	7.74512814	4.74386943	4.67002914
H	7.78905785	5.95446165	5.94542018
H	4.25124936	9.58891115	6.13678326
H	2.98787104	9.64346049	4.91480536
H	6.86754818	3.80159050	6.80266837
H	5.91352162	2.98885514	4.65633858
H	2.11899591	8.69739207	7.04608432
H	1.13162226	7.87810290	4.92788428
H	6.02546593	5.94485545	7.75907243
H	4.25737193	7.73293035	7.86645802
H	0.35415978	6.97940542	7.11439769
H	0.17056854	5.78486165	5.85671877
H	5.16079602	3.79142300	8.64762746
H	3.00082219	2.92213426	7.80447979
H	2.13977558	6.83742057	8.80821150
H	1.16532116	4.75576922	7.88739442
H	4.28959659	5.90552868	9.62842662
H	3.04088530	4.66702343	9.62653394
H	1.93290058	4.02057774	2.05455756
H	2.93913905	6.09009064	1.14125518
H	4.16917012	4.83445903	1.20547350
H	5.04312953	6.98472251	2.11632190
H	0.86887753	5.15584944	3.94050443
H	1.33979637	6.37630435	2.81269660
H	5.09836282	2.04174289	6.80129826
H	4.01815524	3.04701531	2.92362686
H	2.99840290	1.08910034	5.87617228
H	4.21196727	1.23982827	4.61175631
H	1.95522379	2.10147440	3.87198718

H	1.38467846	2.72255414	6.27363864
H	0.87981648	3.90670874	5.12314362

Nonamantane ($C_{34}H_{36}$)

C	3.51671456	5.48838021	1.82970690
C	4.42541905	4.51056184	4.46373088
C	3.64823729	5.28841262	8.97177644
C	6.22332685	6.27841920	4.49247392
C	4.47101903	6.25608021	6.30572338
C	6.24374114	4.46534789	6.21038091
C	7.11797717	5.36667278	5.33537444
C	3.52534585	7.21089930	3.67498701
C	5.36368205	5.30975228	7.14276555
C	3.60661019	8.96033351	5.49241232
C	5.34290948	7.15342177	5.39584951
C	3.56642158	3.56683668	7.12761285
C	5.29851729	5.44525977	3.59378326
C	3.59940650	7.12610862	7.23436018
C	5.31923691	3.60198758	5.34034046
C	4.47749768	4.41989418	8.03062446
C	4.43621198	8.07734973	4.56526812
C	3.53055880	5.38919769	5.40184264
C	3.50866208	3.65204467	3.56831751
C	1.72833296	5.41066908	3.62413149
C	0.86213265	4.38989155	4.37846673
C	0.91559618	6.42354151	6.52479010
C	1.74862263	3.59189883	5.34738930
C	2.73319009	8.07155842	6.37380273
C	2.75425711	6.20573760	8.14177002
C	3.55789402	1.81733843	5.30792180
C	1.77512414	7.20922476	5.52209378
C	4.39132234	6.34633567	2.73901465
C	2.62755549	4.47990616	6.28587503
C	2.60700368	6.31008648	4.55172027
C	4.43240108	2.68921320	6.20396722
C	1.79566251	5.39039961	7.24558169
C	2.64211658	4.58308844	2.69290118
C	2.66300038	2.71760968	4.46045267
H	6.84530805	6.92594302	3.86339118
H	5.91060717	4.82353681	2.92723026
H	5.06242455	8.70365930	3.91919568

H	2.89128165	7.86906961	3.06668247
H	5.98745446	7.77060071	6.03547787
H	7.75574377	4.75806327	4.68549312
H	7.78548885	5.96794562	5.96192261
H	4.26234486	9.58446654	6.10914029
H	2.97742678	9.64110442	4.90896142
H	4.13944633	3.03763800	2.91242090
H	1.09014141	6.06258822	3.01537405
H	6.88036050	3.80962406	6.81589097
H	5.93143171	2.97717234	4.67684434
H	2.12866767	8.70124334	7.03585839
H	1.13700888	7.86792045	4.92061260
H	6.00812179	5.92306669	7.78619720
H	4.26183473	7.73233400	7.86629035
H	2.02611082	2.09608147	3.82142583
H	0.00221039	4.83581837	4.85914544
H	0.44402582	3.69092215	3.64571590
H	2.94314889	1.14314805	5.91383772
H	4.18269266	1.18654985	4.66619875
H	5.07408633	2.05460615	6.82633186
H	1.12494419	2.94123208	5.97235602
H	0.53755177	7.12858284	7.27320301
H	0.03018705	5.99113233	6.07909620
H	5.11835329	3.74850463	8.61402146
H	2.94718179	2.91652539	7.75912260
H	2.14992314	6.82581154	8.81301731
H	1.17223381	4.74672889	7.87797675
H	4.30466158	5.87873977	9.62026416
H	3.03411932	4.66193989	9.62761448
H	2.00515672	3.97086812	2.04498500
H	2.88733497	6.12246765	1.19610663
H	4.14114405	4.88893039	1.15826088
H	5.01847617	7.00966169	2.13193249

4 Convergence with respect to basis sets

Table S1: Tetramantane-like diamond cluster basis set convergence computed using B3LYP and the def2 basis set family.

Basis (H)	Basis (C)	E_d [eV]	Basis Functions
SVP	TZVP no f	7.26	712
SVP	TZVP	7.21	932
TZVP	TZVP no f	6.68	740
TZVP	TZVP	6.64	960
TZVP	QZVP no g	6.54	1224

Tab. S1 show the linear response TDDFT optical gap (E_d) for tetramantane for a range of basis sets in the def2 family. The gap is essentially converged at the TZVP level. Removal of f-functions from carbon atoms has appreciable effect, thus for all breakdown and transient real-time TDDFT simulations, we used def2-TZVP without f-functions for carbons, and def2-TZVP for hydrogen atoms.

5 Range-separated functional tuning

Range-separated hybrid functionals have been shown to reduce the self interaction error common in heavily driven systems such as ours. Tuning of the range-separated functional LC-PBE0 was done via satisfying Koopman's theorem. The amount of HF-exchange and DFT contributions to the short and long range Coulomb potential are α and β, respectively, while ζ is the range-separation attenuation parameter.

$$\frac{1}{r_{12}} = \frac{\alpha + \beta\,\mathrm{erf}(\zeta r_{12})}{r_{12}} + \frac{1 - [\alpha + \beta\,\mathrm{erf}(\zeta r_{12})]}{r\ _{12}} \tag{1}$$

Fig. S3(a) and (b) show the tuning for a coarse and fine grid scan, respectively. The global minimum was determined to be $\alpha = 0.28$, $\zeta = 0.15$ au^{-1}

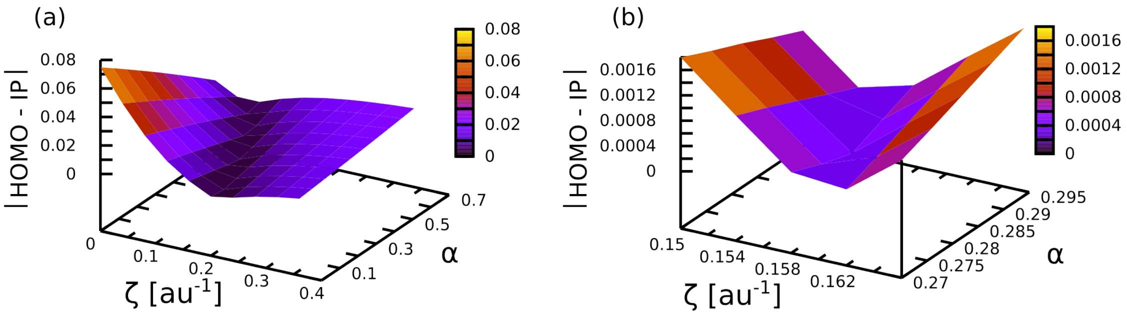

Figure S3: (a) Coarse scan of the ζ and α LC-PBE0 tuning for tetramantane. (b) Finer scan near the global minimum of the target function.

6 Effect of pump polarization

Fig. S4 shows the C K-edge X-ray absorption spectrum for unpumped tetramantane for the x, y, and z polarizations. All spectra were computed with real-time TDDFT using def2-TZVP(-f)/def2-TZVP/TD-LC-PBE0*. The cluster geometry was rotated such that none of the x, y, or z directions lie along a point of symmetry. The three polarizations give very similar spectra due to the homogenous bulk-like nature of our cluster. We selected the x axis for all subsequent calculations.

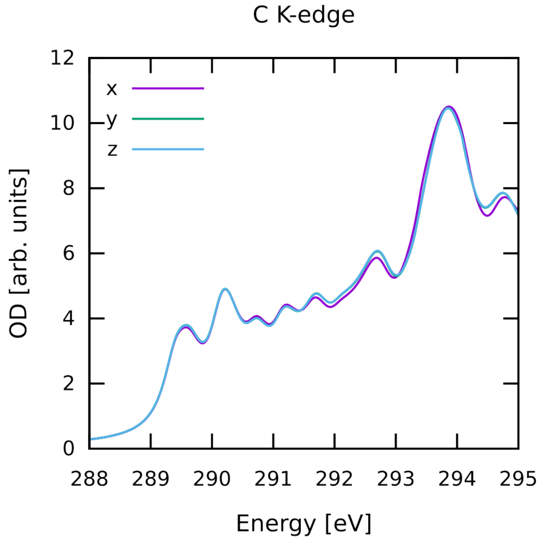

Figure S4: Carbon K-edge X-ray absorption spectrum for unpumped tetramantane computed with RT-TDDFT for three polarizations.

7 Stark-shifting of conduction band

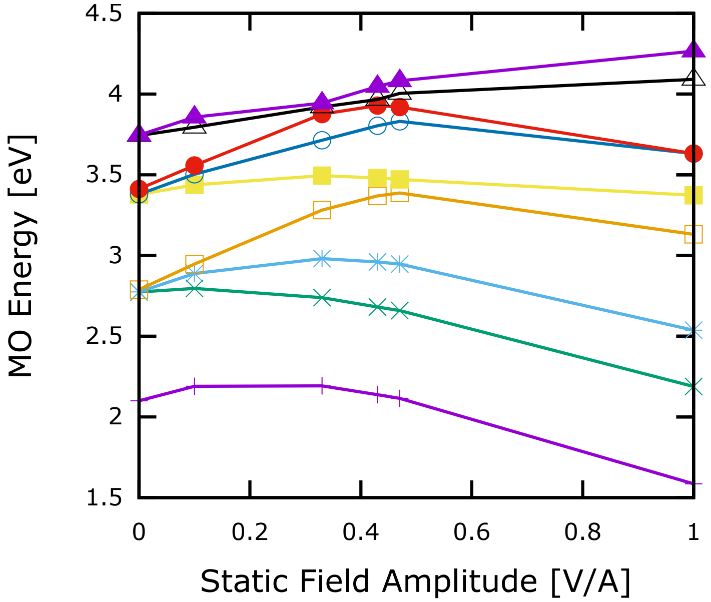

Figure S5: The energy of lowest 9 virtual orbitals are plotted vs static field strength for the diamond ($C_{22}H_{28}$) cluster using LC-PBE0*. As the field increases Stark shifting becomes more prevalent. For reference, breakdown occurs at 0.53 V/Å for this system and DFT functional.

Figure S5 shows the lowest nine virtual MO energy levels calculated with tuned LC-PBE0* with static fields applied between 0 and 1 V/A. As determined via LR-TDDFT, the lowest state (purple pluses) does not contribute significantly to Peak A but is shown for completeness. Fig. S6 shows the corresponding C K-edge LR-TDDFT (up to 50 roots). The MO energies shift with field amplitude, and begin to cross over (switch character) for field amplitudes greater than 0.3 V/A. At the maximum amplitude at which we pump our system (0.47 V/A), the three nearly degenerate CB states that give rise the first peak in Fig. S6 are split by 0.74 eV, and the three states involved in the second peak are split by 0.51 eV. Collectively, these result in a broadened and shifted LR spectrum and a roughly 30% modulation of the OD at Peak A. Note that in this analysis, energies are self-consistently converged with a static field applied, meaning that the shifts observed here are larger than would be observed with an alternating field and provide an upper limit to the shift. That

being said, the observed oscillations that we observe are at least consistently with Stark effects at the pump amplitude.

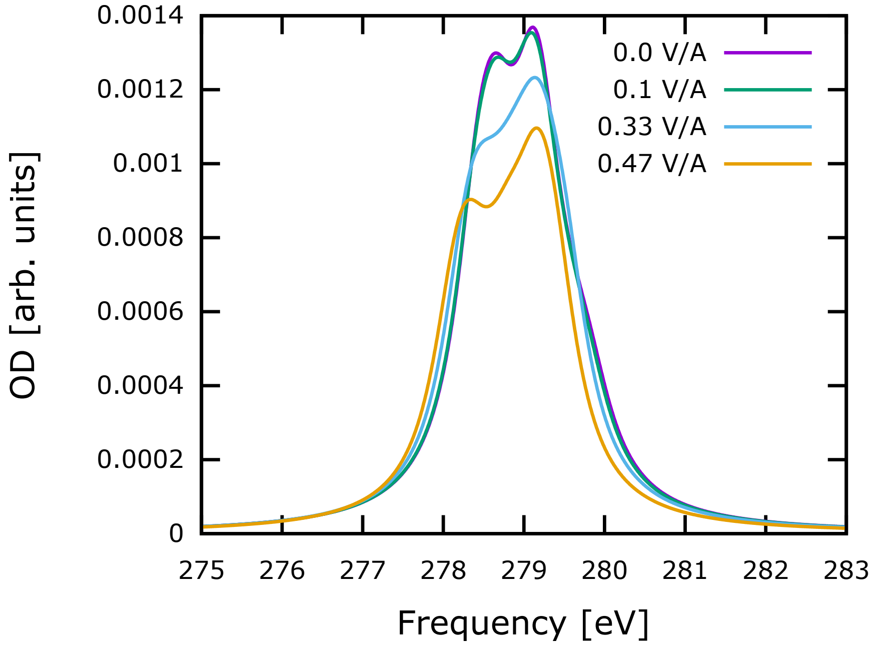

Figure S6: The first 50 roots of the carbon K-edge linear-response TDDFT spectra for a range of static field amplitudes. The peaks broaden and slightly red-shift with increasing field amplitude due to the Stark shifting of the states involved in the transitions.

8 Fitting adiabatic population

To decompose the occupations of the states into population and polarization (Stark) contributions, the zero crossings of the electric field were fitted to the envelope's function. At $E = 0$, the instantaneous Stark effect is zero. Each orbital was the fitted with parameters a and t_0 to the Hann envelope function:

$$f(t) = a \sin^2 \left(\frac{\pi(t - t_0)}{w_P} \right) \quad (2)$$

The width w_P was chosen to be that of the pump field and not fitted whereas the pulse center t_0 was allowed to vary. Fig. S7 shows and example of this fitting for the case of orbitals 75 and 85.

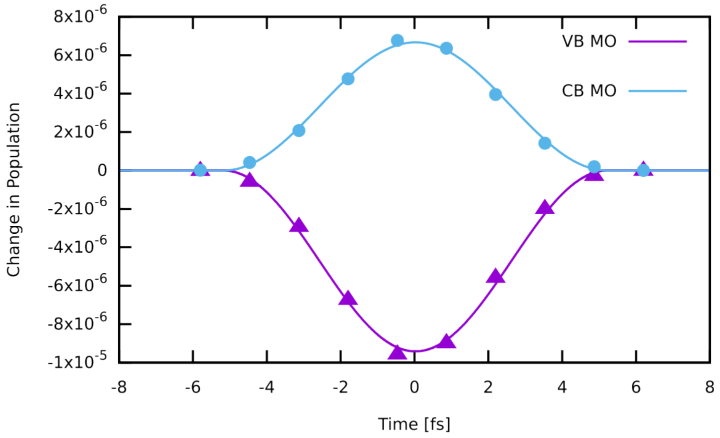

Figure S7: Populations at times when the electric field is zero, and the corresponding fit to the envelope of the field, for the specific examples of orbital 75 (valence band; HOMO-5) and orbital 85 (conduction band; LUMO+4). Fitting the zero field values to the field envelope provides an approximate measure of the VB hole and CB electron populations without polarization effects.

References

(1) Yang, M.; Sissay, A.; Chen, M.; Lopata, K. Intruder Peak-Free Transient Inner-Shell Spectra Using Real-Time Simulations. *J. Chem. Theory Comput.* **2022**, *18*, 992–1002.
